# Early Employment Outcomes in Autistic and Non-autistic Youth: Challenges and Opportunities

**DOI:** 10.1007/s10803-025-07001-9

**Published:** 2025-08-07

**Authors:** Jo A. Yon-Hernández, Catherine Gonzales, Surina Bothra, Kali Kecskemeti, Ana-Maria Iosif, Yukari Takarae, Steve Ruder, Susan R. McGurk, Kim T. Mueser, Marjorie Solomon

**Affiliations:** 1 Department of Psychiatry and Behavioral Sciences, MIND Institute, University of California, Davis, 2825 50th Street, Sacramento, CA 95817, USA; 2 Public Health Sciences, University of California Davis, Davis, CA, USA; 3 MIND Institute, University of California Davis, Sacramento, CA, USA; 4 Department of Occupational Therapy; Department of Psychological and Brain Sciences; Center for Psychiatric Rehabilitation, Boston University, Boston, MA, USA

**Keywords:** Youth employment, Executive functioning, Supported employment, Transition to employment

## Abstract

Autistic youth often encounter significant barriers in securing employment, including difficulties with job acquisition, limited workplace support, and reduced access to structured employment services. This study examined early employment experiences in cognitively able autistic and non-autistic youth, with a focus on job characteristics and the associated factors of employment status. Participants included 99 individuals (51 autistic, 48 non-autistic) aged 18–23. Open-ended responses were coded to characterize first job experiences, including job setting, duration, hours worked, support received, sector, and job acquisition method. Group differences were assessed using chi-square tests. Logistic regression was used to examine the predictors of employment outcomes, including IQ, executive functioning, adaptive functioning, and education level. Results revealed notable differences between groups, with 67% of autistic participants having had a first work experience compared to 86% of non-autistic participants. When unpaid experiences (such as WorkAbility/internships) were excluded, this gap widened to 50% versus 78%. Autistic participants were significantly less likely to obtain jobs through competitive hiring and were more likely to work in sales/retail-related roles, whereas non-autistic participants exhibited greater job diversity and career-oriented positions. Personal connections were critical to job acquisition for autistic individuals, although structured employment programs were also a key pathway. Executive functioning difficulties were significantly associated with lower employment likelihood. Early employment disparities persist among autistic youth, particularly in access to competitive and career-track jobs. Interventions that support executive functioning, expand structured employment options, and leverage family and social networks may enhance employment success during the transition to adulthood.

## Introduction

Autistic individuals face significant challenges in accomplishing typical adult goals, such as pursuing higher education to start professional careers (White et al., 2024), developing mature social and community relationships, and securing steady employment (Cheak-Zamora & Teti, 2015). Their professional journeys are also marked by distinct obstacles, including a lack of transition services, interview difficulties, inadequate workplace supports, misconceptions about their unique needs and behaviors, a lack of adequate services to support them on the job, and discrimination, (Harmuth et al., 2018; Sarret, 2017). However, there is a lack of comprehensive research investigating the work experiences of autistic youth^1^.

Workforce participation plays a pivotal role in enhancing the overall life satisfaction and well-being of autistic individuals (Wehman et al., 2014, 2017). Research also has identified a strong relationship between work and mental and physical health (Brouwers et al., 2023). In addition to necessary income, employment can provide autistic individuals with important opportunities for social connections, personal growth, and a sense of purpose (Dunstan et al., 2017). Despite these beneficial outcomes, employment rates for people with autism has not increased significantly from the rates a decade ago (Taylor & Seltzer, 2011; Wehman et al., 2014). Although employment rates vary by region and population characteristics, various studies estimate that only 15–23% of autistic individuals in the U.S. are employed (Bureau of Labor Statistics, 2025; Hickey et al., 2024). Relevant to this study, the California State Council on Developmental Disabilities (2023) reported that only 13.8% of adults with developmental disabilities were employed. Data suggest high unemployment rates in autistic people and highlight the need to remedy this problem because of the negative consequences of unemployment.

### Challenges in Obtaining Employment for Autistic Youth

Autistic youth face a “services cliff,” whereby state-funded services related to the educational system terminate as high school graduation approaches, just when they need the most support in transitioning to adult activities. For example, California has hosted the well-established WorkAbility I (WAI) program since 1982 (California Department of Education, 2023)^2^. WAI is explicitly designed to prepare students with disabilities for the labor market. It offers comprehensive pre-employment skills training, facilitates employment placement in a setting with other non-disabled workers, and provides ongoing support for high school students in special education who are transitioning from school to work, to independent living, and to postsecondary education or training. However, the long-term efficacy of WAI in securing and sustaining competitive integrated employment (CIE) (employment in an integrated community setting that pays at least minimum wage and includes employee benefits commensurate with those of other workers) remains uncertain. Furthermore, only a small percentage of students receive these services.

Although federal and state employment assistance programs exist across the U.S. to support individuals with disabilities—such as services provided through State Vocational Rehabilitation (VR) agencies, Developmental Disabilities councils or boards, and various regional providers—there are significant barriers to receiving these supports, including the complexity involved in accessing them, restrictive qualification requirements (Solomon et al., 2023), inconsistencies in eligibility criteria across agencies and states, limited available services in many localities (Roux et al., 2023; Solomon et al., 2023), and the fact that some services are eliminated in times of funding shortfalls. For instance, in California—where the current study was conducted—services are typically offered through the Department of Rehabilitation (DoR) and the Department of Developmental Services (DDS), which operate through Regional Centers (RCs). While these agencies provide crucial support, they are not universally accessible and often vary in terms of capacity, quality, and eligibility requirements. In fact, Roux et al. (2015) indicated that approximately one-fourth of autistic adults in the U.S. remain disconnected from available employment services. In addition, many individuals seek to forgo employment because of fears related to losing Social Security entitlements, such as the Supplemental Security Income (SSI) and Social Security Disability Insurance (SSDI) for individuals earning below certain thresholds. This issue is not merely a matter of personal preference but often stems from financial necessity, as many autistic individuals work in part-time or low-wage positions that do not offer employer-sponsored health insurance or other critical benefits. As a result, sustained reliance on public assistance remains essential for ensuring financial security and access to healthcare benefits provided by the state and federal governments.

Once a job is obtained, another major challenge is receiving adequate on-the-job support. Although autistic employees often possess unique skills that can be assets in their work efforts, such as exceptional attention to detail, pattern recognition abilities, and intense focus (Bury et al., 2020; Cope & Remington, 2022); little research has focused on how to effectively support them on the job (Krzeminska et al., 2019). Literature from successful transitional employment programs, such as Project SEARCH +ASD, has identified effective supports, such as embedded job coaching, the use of visual supports, and individualized goal setting within inclusive work environments (Wehman et al., 2014, 2020). Another evidence-based approach proven to be the most effective way to support autistic job seekers is supported employment. This approach provides one-on-one assistance to help individuals with disabilities get and keep jobs in the community (Howlin et al., 2005; Lynas, 2014; Martin & Lanovaz, 2021). This model typically includes personalized support such as job coaching, workplace accommodations, and ongoing assistance tailored to the individual’s needs, typically provided by an employment specialist (Howlin et al., 2005; Lynas, 2014; Martin & Lanovaz, 2021; Schall et al., 2020). However, this level of support is not widely available to most autistic individuals. As a result, negative attitudes from coworkers or supervisors can increase pre-existing social anxiety in autistic employees (Briel & Getzel, 2014), potentially deterring them from requesting the necessary accommodations or support that would enable them to demonstrate the ability, motivation, and skills to perform well in suitable roles that align with both their strengths and employer’s needs (Hendricks, 2010). This underscores the importance of comprehensive vocational support to facilitate successful job matching and the creation of an environment in which autistic employees can succeed.

### Other Factors Associated with Employment in Autism

Empirical studies suggest that other factors may influence job acquisition and retention. Adaptive functioning is defined as an individual’s ability to meet the demands of daily living, including behaving appropriately in a work setting and taking care of one’s own adult social relationships, living arrangements, finances and health (Hernández-Finch et al., 2023; Kanne et al., 2011), which could influence employment outcomes. Better adaptive functioning has been positively associated with vocational outcomes in autism (Hernández-Finch et al., 2023; Roux et al., 2013); lower levels of adaptive behavior have been associated with unemployment in autistic individuals (Hernández-Finch et al., 2023). Another study found that autistic individuals had greater difficulty selecting appropriate social responses to workplace scenarios (Scott et al., 2018).

IQ has also been positively associated with both employment status and job performance. Research examining the strength of this association in the general population has shown that IQ predicts from 4 to 30% of variance in job performance (Richardson & Norgate, 2015; Sternberg et al., 2001). Studies following children with neurodevelopmental disorders, including autism, through the transition to adulthood found that participants with above-average verbal intelligence quotient (VIQ) were more likely to secure employment (Lord et al., 2020a, b), and that CIE is three times more likely in autistic individuals without an intellectual disability (ID) (Hickey et al., 2024; Taylor & Seltzer, 2011). Autistic children with an IQ > 70 generally have better adult outcomes than those with an IQ below 70 (Howlin et al., 2004). Individuals with lower IQs within the average range are less likely to be employed than those with higher IQs, underscoring that cognitive abilities play a critical role not only in obtaining employment but also in maintaining successful job performance (Hickey et al., 2024; Lord et al., 2020a, 2020b). Nonetheless, because functional outcomes are significantly variable among autistic individuals without ID, we should exercise caution in using IQ as a clinical tool to predict individual outcomes (Holwerda et al., 2012).

Challenges in various aspects of executive functioning, including working memory, inhibitory control, cognitive flexibility, planning, and self-awareness, can be prominent in autism (Demetriou et al., 2021; van den Bergh et al., 2014). These difficulties may also lead to job-related social problems, lower job satisfaction, and poorer work outcomes (Woolard et al., 2021), and are closely linked to challenges in adaptive functioning skills, which are essential for navigating the demands of employment. Research has increasingly highlighted that autistic individuals often experience executive functioning challenges in the workplace, such as difficulties with self-management, problem-solving, adapting to change, and navigating complex social environments. These impairments can interfere with understanding social cues, following instructions, and adjusting to implicit workplace norms (Grainger et al., 2016; Hendricks, 2010; McMahon et al., 2016). As a result, individuals may exhibit inappropriate behavior, require more explicit guidance, and struggle with both job acquisition and long-term retention.

This study describes the challenges and opportunities that autistic youth encounter when transitioning from high school to the workforce during the age 18 to 23 age period (by the time participants in special education finish high school). Given the critical nature of this time in which individuals transition from formal education to gaining hands-on experience in the workforce and begin to develop a career path, we examined the following questions:

1. What proportion of autistic youth, compared to non-autistic youth, have experienced work by age 23. Based on prior research highlighting disparities in vocational development trajectories (Hickey et al., 2024; Roux et al., 2013), we hypothesized that non-autistic individuals would be more likely to secure their first work experience during this time than their autistic peers.
2. Do the first work experiences of autistic individuals differ from non-autistic individuals with respect to job setting, duration, weekly hours worked, and employment support provided? Here we hypothesized that autistic individuals would have shorter job durations, fewer work hours (Ohl et al., 2017), and a greater inclination towards working in a narrower range of lower-skilled jobs, while non-autistic individuals would hold a wider variety of job occupations.
3. To what extent do autistic individuals access state-funded employment services (e.g., WAI Program, RC or DDS services, DoR), and are these services associated with securing and retaining employment, particularly in first jobs?
4. Does the likelihood of employment among autistic individuals depend on factors such as adaptive functioning, IQ, and executive functioning skills? Drawing from previous research (Hernández-Finch et al., 2023; Howlin et al., 2004; Woolard et al., 2021), we hypothesized that these variables would significantly contribute to the employment status of autistic individuals.

## Methods

### Participants

This research was conducted as part of a 5-year cohort sequential study (Prinzie & Onghena, 2005) that examined cognitive development during the transition to adulthood at three assessment timepoints from adolescence to young adulthood (Cognitive Control in Autism Study [CoCoA]; R01 MH106518). Autistic and non-autistic participants were recruited from the greater Sacramento area using print and online advertisements, physician referrals, and volunteer registry/subject tracking systems. For a full description of the recruitment process and the assessments used, please refer to Solomon et. al. (2021). The University of California Davis Institutional Review Board approved the research protocol, and written informed consent was obtained from all participants. The complete cohort included 176 participants (88 non-autistic and 88 autistic). Participants in both groups met the established criterion of having an Intelligence Quotient (IQ) of ≥ 70 as determined by the Wechsler Abbreviated Scale of Intelligence, Second Edition (Wechsler, 2011). Additionally, given the fact that California labor laws impose stringent regulations on the employment of minors (under the age of 18) (DIR, 2020), we reasoned that participants under the age of 18 would confound results, so they were excluded. The total sample for the current study consisted of 99 participants [autistic = 51 (11 female); non-autistic = 48 (11 female)]. Both the autistic and non-autistic groups ranged in age from 18 to 23 years. Although the original study aimed to match participants on age, assigned sex at birth, and IQ, for this subsample, we included only individuals who met the study criteria—specifically, those aged 18–23 who completed the questionnaires.

We confirmed the diagnoses of all members of the autistic group using the gold-standard Autism Diagnostic Observation Schedule, Second Edition (Lord et al., 2000). Among the participants, 14 participants (29%) were still enrolled in high school, 12 participants (25%) had completed high school, while 22 individuals (46%) were either currently enrolled in or about to begin post-secondary education or had completed it. This included enrollment in traditional college programs as well as vocational or technical education. Four participants (9%) reported a diagnosis of attention deficit hyperactivity disorder (ADHD), 1 (3%) reported an anxiety disorder for which they were actively taking medication, and 1 (3%) reported experiencing depression.

In the non-autistic group, 3 participants (6%) were still enrolled in high school, and 6 participants (12%) had completed high school, while 42 individuals (82%) pursued post-secondary education. No co-occurring conditions or medication use were reported by non-autistic participants. For a complete description of the sample demographics, see Table 1.

### Measures

#### Wechsler Abbreviated Scale of Intelligence, 2nd Edition (WASI-II) (Wechsler, 2011)

The WASI-II (Wechsler, 2011) is a standardized cognitive assessment for individuals aged 6–90 years. It provides full-scale IQ (FSIQ) derived from verbal IQ (VIQ) and Performance IQ (PIQ) scores. Scores range from 40 to 160, with a mean of 100 and a standard deviation of 15. In this study, FSIQ, VIQ, and PIQ were used as independent variables.

#### Adaptive Behavior Assessment System, Third Edition (ABAS-3) (Harrison, 2015)

The ABAS-3 is a comprehensive tool for evaluating adaptive behavior and related skills across lifespans. The questionnaire allows raters to indicate the frequency of an individual’s performance on many activities on a 4-point scale. In this study, we used the adult self-report form (Yon-Hernández et al., 2025). The ABAS-3 provides a General Adaptive Composite (GAC) score, which encompasses the conceptual, social, and practical domains of adaptive functioning. This tool is well-suited to individuals with intellectual abilities greater than 70 because it covers more advanced adaptive skills. The GAC score was used as an independent variable.

#### The Behavior Rating Inventory of Executive Function-Adults (BRIEF2A) (Roth et al., 2005)

The BRIEF2A is a standardized tool for screening executive function-related difficulties in individuals aged 18 years and older. It includes three indices: behavioral control index, emotional control index, and cognition regulation index, which together compose the General Executive Function (GEF) score. Higher scores are associated with greater impairments, with scores from 60 to 64 considered mildly elevated, 65 to 69 potentially clinically elevated, and scores above 70 clinically elevated. GEF scores were used as independent variables.

#### Global Functioning: Role Scale (GF: Role) (Carrión et al., 2018; Cornblatt et al., 2015)

At each timepoint, the participants completed the GF: Role scale. This instrument was administered as a structured interview and includes open-ended questions and evaluates quantity and quality of role functioning depending on age. In addition to age appropriateness, ratings are based on role demands, level of independence or support provided (ranging from full independence to increasing levels of monitoring and guidance), and overall performance within the role given the level of support. The GF: Role was designed to be used by experienced clinicians/researchers for direct interviews guided by the accompanying probes. The GF: Role scale has been validated in youth at high risk of schizophrenia (Cornblatt et al., 2015). For this study, we adapted the scale to better capture autism-specific social and role challenges and ensure age-appropriateness. The revisions included refining prompts and anchors to inquire about unpaid work experiences because many individuals engage in such activities during this period. We also incorporated questions about job settings, types of employment support received, and methods of job acquisition. Additionally, we adapted the school-related prompts to determine whether the participants were still completing high school—recognizing that autistic individuals may take longer than average to graduate—and whether they were pursuing postsecondary education (technical training, community college, four-year college and/or plans about getting more advanced training after college graduation). We also inquired about whether they were simultaneously working and studying. Furthermore, we integrated modifications to reflect technology-based interactions, such as social media and online communication, and shifted the focus from generalized psychosis-related clinical symptoms (e.g., paranoia). These adaptations were informed by expert consensus of members of our University Center of Excellence in Developmental Disabilities to enhance the scale’s relevance and utility for the study population.

The original longitudinal study included three assessment timepoints, with data collection occurring when participants were between the ages of 12 and 22. Because this study used a sequential cohort design with ongoing recruitment, participants completed assessments at different timepoints. For the present analysis, we interviewed and collected employment data from any visit in which the participant was between 18 and 23 years old, regardless of whether it was their first, second, or third assessment. Open-ended responses were systematically coded using a structured framework developed from common themes identified in the initial participant responses. Two independent coders reviewed and categorized the responses, resolving discrepancies through a consensus process to ensure reliability and accuracy in capturing the nuances of participants’ employment experiences. A detailed description of the adaptation process and coding scheme is provided in the Supplemental Information. GF: Role-scale responses were used as independent variables.

### Analysis

Descriptive statistics were used to characterize the participants’ demographic characteristics, assessment scores, and work experiences. Work experiences included both unpaid experiences (e.g., internships or WAI experiences) and paid work experiences, which encompassed part-time, full-time, and seasonal jobs. Some participants reported more than one work experience (Table 1). However, although we report number of jobs held/individual and include this information in regression analyses for all group comparisons, only the participant’s first job experience was used to compare between groups. To analyze group differences (Autistic vs. Non-Autistic), we used independent *t*-tests for our continuous variables (i.e., age, FSIQ, VIQ, PIQ, GEF, GAC). The chi-square test of independence was used to compare both groups for all categorical variables (i.e., number of jobs held, employment status, job acquisition method, employment support and sector of job, job duration and weekly work hours).

To examine whether diagnosis (i.e., being autistic or non-autistic), FSIQ, VIQ, PIQ, adaptive functioning, and executive functioning were associated with the employment status (i.e., unemployed vs. employed), we conducted two separate logistic regression analyses. The first logistic regression used employment status as the dependent variable, including all work experiences (both paid and unpaid, such as internships and WAI program experiences). The second logistic regression applied a stricter definition of employment status, considering only CIE opportunities and excluding all unpaid work experiences. In both models, diagnosis (autistic vs. non-autistic) was entered as a predictor, allowing us to examine the effect of diagnostic status on employment outcomes. For both models, we conducted a second step in which we added age and education level (high school vs. post-secondary enrollment) to assess their additional contribution to employment outcomes. In both models, diagnosis (autistic vs. non-autistic) was entered as a predictor, allowing us to examine the effect of diagnostic status on employment outcomes. The analyses for this paper were generated using SAS Version 9.4 (SAS Institute Inc., 2021).

## Results

### First Work Experiences in Autistic and Non-Autistic Youth by Age 23

A sample summary is provided in Table 1. The descriptive statistics indicate that 67% of autistic individuals and 86% of non-autistic individuals in our sample had participated in at least one work experience between the ages of 18–23. When unpaid first work experiences (e.g., unpaid internships or work experiences through the WAI program for autistic individuals) were excluded, the proportion decreased to 50% for autistic participants and 78% for non-autistic individuals. The average age at first job experience was 19.4 years (SD = 1.6) for autistic and 19.9 years (SD = 1.5) for non-autistic individuals; excluding internships, the averages were 19.7 (SD = 1.6) and 19.9 (SD = 1.5), respectively.

The chi-square test of independence was conducted to examine whether the likelihood of acquiring first work experience differed between groups. The results indicated a significant association between autism diagnosis and first work experience, indicating that the autism group had participated in fewer experiences, χ^2^(1) = 5.330, *p* =.021, Cramér’s V = 0.23, indicating a moderate effect. Similar results were found when unpaid work experiences were removed, χ^2^(1) = 8.746, *p* =.003, Cramér’s V = 0.30, also indicating a moderate effect.

### Characteristics of First Work Experiences Across Groups

A series of Chi-Square tests were conducted to examine group differences in first work experiences with respect to inclusivity of job setting, employment duration, weekly hours worked/week, whether employment support was present or not, job sector, and job acquisition method. In addition, descriptive statistics (n, %) were used to specific types and patterns of work experiences withing each group, as shown in Table 2.

### Job Setting

A significant difference was found in job setting between the groups, χ^2^(1) = 3.920, *p* = 0.048, Cramér’s V = 0.223, indicating a small effect size. The majority of both groups worked in fully inclusive settings, with 90% of autistic individuals and 100% of non-autistic individuals having this experience.

### Employment Support

There was a statistically significant difference in employment support between autistic and non-autistic individuals, χ^2^(3) = 11.190, *p* = 0.011, Cramér’s V = 0.376, indicating a moderate effect size. 77% of autistic individuals worked independently, compared to 100% of non-autistic individuals. Some autistic individuals received employment support through group employment classes (7%), job coaching (13%), or other support such as assistance from a supervisor (3%). However, no non-autistic individuals reported receiving these types of support, which is consistent with the fact that non-autistic adults often do not meet the eligibility criteria for funded employment programs typically designed for individuals with disabilities.

### First Job Duration

There were no significant group differences in job duration, χ^2^(7) = 6.860, *p* = 0.444, Cramér’s V = 0.307. However, among those employed, autistic individuals were more likely to have shorter job durations. 33% had jobs lasting 2–3 months, compared to 16% of non-autistic individuals. 7% of autistic individuals worked for 1 year, compared to 14% of non-autistic individuals. 23% of autistic individuals had jobs lasting more than 1 year, compared to 35% of non-autistic individuals.

### Weekly Work Hours

No significant differences were found in weekly work hours, χ^2^(6) = 10.200, *p* = 0.116, Cramér’s V = 0.364. Autistic individuals were more likely to work fewer hours: 32% worked less than 10 h per week, compared to 19% of non-autistic individuals. Only 3% of autistic individuals worked full-time (40 + hours) compared with 17% of non-autistic individuals.

### Job Sector

A significant difference was found in the job sector, χ^2^(13) = 22.868, *p* = 0.043, Cramér’s V = 0.549, suggesting a large effect size. A majority of autistic individuals were employed in what are typically entry-level jobs in retail establishments (52%; such as supermarkets stocking shelves or bagging), compared to only 14% of non-autistic individuals. Non-autistic individuals exhibited a wider distribution of types of employment having entry-level jobs such as in Food Services (21%) and in Sales/Retail (14%). As well as a wider career-track jobs, with higher representation in Life, Physical, and Social Sciences (12%), Arts, Design, Entertainment, and Media (12%), and Education (14%).

### Job Acquisition Method

A significant difference was found in how participants obtained their first job, χ^2^(3) = 19.452, *p* = 0.001, Cramér’s V = 0.496, indicating a large effect size. Both groups used personal connections to obtain a job (10% autistic vs. 5% non-autistic individuals). Autistic individuals were significantly less likely to obtain jobs through competitive hiring (48% vs. 95% of non-autistic individuals). Autistic individuals were more likely to secure jobs through the WAI program (26%) and supported employment services (16%).

### Number of Jobs Held

While chi-square analyses were conducted for only participants’ first work experience due to sufficient sample size and a more even distribution across job categories. For later experiences (second, third, and fourth), too few participants reported these jobs, or the data were too unevenly distributed to support valid statistical comparisons. Descriptive results are presented in Table 2 to illustrate potential trends over time. Across all work experiences, both autistic and non-autistic individuals primarily worked in fully inclusive settings. Autistic participants consistently held part-time positions, often working fewer than 20 h per week, and tended to remain concentrated in a narrower range of entry-level jobs, particularly sales/retail and food services. In contrast, non-autistic participants showed a gradual shift toward a broader variety of job sectors over time, including more career-oriented fields such as healthcare and engineering. Over time, both groups increasingly obtained jobs through a competitive hiring process.

### Access To and Outcomes of State-Funded Employment Services

Relatively few autistic participants in this study received state-funded employment services. Of the 52 autistic individuals, 7 reported participating in the WAI program (8%), more than one time during the end of their high school years, but none reported securing sustained employment following program completion. Additionally, 10 participants received other services from the RC/DDS, and 8 received services from the DoR. Of these, 8 individuals were concurrently enrolled in both RC and DoR services, but only 5 reported obtaining a job without using the WAI pathway through these services. Furthermore, only 2 participants were beneficiaries of the SSI.

### Factors Associated with Employment Status Among Autistic Youth

The first logistic regression analysis (which included only the first work experiences including unpaid and paid experiences), revealed limited predictive ability. Model_1_, which included diagnosis, FSIQ, VIQ, PIQ, GAC, and GEF, was not statistically significant, χ^2^(6) = 8.730, *p* = 0.189, indicating that these predictors alone did not significantly explain the variance in whether participants were employed or unemployed. The addition of age and education level in Model_2_ slightly improved model fit but did not reach statistical significance, χ^2^(2) = 3.587, *p* = 0.166. The model fit indices suggest weak explanatory power, with a McFadden’s R^2^ of 0.119, Nagelkerke’s R^2^ of 0.183, and Cox & Snell’s R^2^ of 0.120 (see Table 3).

The second logistic regression analysis focused on strict employment outcomes (i.e., only paid CIE experiences) revealed limited predictive utility. Model 1, which included diagnosis, FSIQ, VIQ, PIQ, GAC, and GEF, was statistically significant, χ^2^(6) = 19.129, *p* = 0.004, and demonstrated moderate explanatory power (McFadden’s R^2^ = 0.157, Nagelkerke’s R^2^ = 0.254, Cox & Snell’s R^2^ = 0.186). The addition of age and education level in Model 2 further improved model fit and remained statistically significant, χ^2^(2) = 10.435, *p* = 0.005, with increased explanatory value (McFadden’s R^2^ = 0.242, Nagelkerke’s R^2^ = 0.373, Cox & Snell’s R^2^ = 0.272) (see Table 3). Among the individual predictors, higher executive functioning difficulties were significantly associated with employment status in both models (Model_1_: OR = 1.069, 95% CI [1.011, 1.123], *p* = 0.019; Model_2_: OR = 1.069, 95% CI [1.018, 1.133], *p* = 0.011), suggesting that greater executive difficulties were linked to poorer employment status. Furthermore, adaptive functioning was a significant predictor in Model_1_ (OR = 1.054, 95% CI [1.010, 1.094], *p* = 0.015), indicating that better adaptive functioning was associated with higher employment likelihood, although this effect was no longer significant in Model_2_ (*p* = 0.085). Education level also emerged as a significant predictor in Model_2_ (OR = 5.972, 95% CI [1.626, 2.947], *p* = 0.003), with individuals with postsecondary education being more likely to be employed (see Table 4).

## Discussion

### First Work Experiences in Autistic and Non-Autistic Youth by Age 23

The current study examined the vocational experiences and potential determinants of job outcomes for cognitively able autistic youth compared to their non-autistic peers. Our findings reveal significant disparities in first work experiences, job settings, employment supports, and employment acquisition methods between these groups. While 67% of autistic individuals reported having had a first work experience between the age of 18 to 23, this was significantly lower than the 86% of non-autistic individuals. When unpaid work experiences were excluded, this gap widened, with 50% of autistic participants having had a first paid work experience compared to 78% of their non-autistic counterparts. Notably, autistic individuals were significantly less likely to obtain jobs through competitive hiring processes (48% vs. 95%) and more likely to secure employment through state-funded employment programs (16%). Job settings also differed, with autistic individuals more frequently employed in low level retail positions such as stocking shelves and bagging groceries, whereas non-autistic individuals had a wider variety of job placements, including roles in customer service, healthcare, science, engineering, media and entertainment, food service, and administrative support. While work hours did not significantly differ between groups, autistic participants were more likely to work fewer hours per week, and only 3% worked full-time compared to 17% of non-autistic individuals. Additionally, only a small subset of autistic participants (9 out of 52) received state-funded employment services. Logistic regression analyses further indicated that executive functioning difficulties and education level were significant predictors of employment outcomes, with higher executive functioning difficulties associated with lower employment likelihood and post-secondary education associated with greater employment success. These findings underscore the challenges autistic youth face in securing meaningful employment and highlight the need for expanded employment services (not only to provide internship opportunities), targeted supports, and structured pathways to help autistic individuals to find and keep jobs.

### Characteristics of First Work Experiences Across Groups

We observed notable differences in work hours between the two groups. While 7 (16%) non-autistic individuals worked full-time (40 + hours per week), only 1 (3%) autistic participant did. The autistic participants displayed considerable variability in their work hours, with the majority working fewer than 30 h per week, suggesting that they predominantly held part-time roles. This aligns with previous studies showing that autistic young adults are often offered fewer than 30 h of work per week (Ohl et al., 2017). Only 9 out of 31 autistic participants reported working 20–39 h per week, while 11 worked between 10 and 19 h, and the majority of individuals 10 (32%) worked less than 10 h. In contrast, 31% of the non-autistic participants had part-time positions and worked between 20 and 39 h, and 17% had held full-time roles that involved working 40 or more hours. Many participants in both groups were also enrolled in school, which may have influenced the number of hours they could work. Given that 82% of non-autistic individuals and 48% of autistic individuals pursue postsecondary education, academic commitment likely shaped their work availability. However, despite similar educational demands, non-autistic participants obtained their first job earlier, worked more hours from the outset, and had longer job tenures than their autistic counterparts. Although we did not assess whether the participants actively sought part-time or full-time employment, early engagement in work experiences, regardless of number of working hours, is crucial for improving employment outcomes in adulthood, particularly for autistic individuals (Black et al., 2020). It is also important to recognize that for some autistic individuals, working fewer hours or focusing on school alone may reflect a deliberate and adaptive choice rather than a limitation. Further research is warranted to examine how this approach may benefit autistic workers.

Our results showed that 53% of the autistic sample were employed in the sales/retail sector, primarily stocking shelves and bagging groceries, compared to only 14% of the non-autistic participants. In contrast, non-autistic individuals exhibited employment across a broader distribution of job sectors and were more likely to hold career-oriented positions, with higher representation in fields such as life, physical, and social sciences (12%), and arts, design, entertainment, and media (12%). This suggests that while autistic individuals tend to work entry-level jobs in roles with structured environments and predictable routines—features that may align with their preferences and strengths (Cope & Remington, 2022; Petty et al., 2023)—non-autistic individuals are more frequently employed in roles that provide greater opportunities for career advancement. However, it is important to acknowledge the diversity of preferences and abilities among autistic individuals and ensure they have access to a variety of job opportunities beyond entry-level retail positions if that is not their preference. Many autistic individuals possess skills that are highly valuable in the technology, engineering, and research industries (Costello et al., 2021; Hillier et al., 2007).

Despite the existing literature advocating for various support mechanisms, such as accommodations, mentors, job coaches, and peer support systems (Anderson et al., 2021; Hagner & Cooney, 2005; Hillier et al., 2007), our study revealed a glaring absence of such support, with 77% of autistic participants working independently without any form of assistance. While some autistic individuals received support through group employment classes (7%), job coaching (13%), or other forms of assistance such as tutoring with a supervisor (3%), these resources were used by only a small fraction of the sample. This lack of support may have contributed to employment challenges, particularly given the high job instability observed among autistic participants, with many experiencing short job tenures. Although employment services can provide meaningful support, their limited utilization, effectiveness, and availability may have intensified the employment challenges observed in this study.

A noteworthy finding of this study concerns the significance of personal networks in the labor market. A large number of autistic participants in our sample obtained employment through personal connections. This finding aligns with Hillier et al. (2007), who also observed that autistic individuals often find employment through personal or family connections. Securing employment through personal networks is also common among non-autistic individuals (Gee et al., 2017), especially those with other disabilities (Khare et al., 2020) and those seeking jobs for the first time (Tholen et al., 2013). Indeed, a recent study found that 65% of individuals with severe mental illness obtained a work position through family, friends, or social contacts (Khare et al., 2020, 2021). It is thus important to acknowledge that parental involvement in employment helps autistic adults leverage personal connections and is just as beneficial for them as it is for non-autistic individuals. Notably, in our sample, securing employment through personal connections (3, 10%) was just as effective for the autistic group as it was for the non-autistic (2, 5%). Highlighting the critical role that social networks play in job acquisition for youth. This finding underscores the need for employment interventions that incorporate strategies that actively engage families and social networks in the job search process.

### Access To and Outcomes of State-Funded Employment Services

Our findings align with studies showing low enrollment of autistic individuals in supported employment services (Anderson et al., 2021; Brouwers et al., 2023; Roux et al., 2023). Only a small portion of the participants in the current study accessed state-funded employment services. Accessing these services may be particularly difficult for those without co-occurring intellectual disabilities who are considered cognitively able and may not fully qualify for them (Solomon et al., 2023), such as could have been the case for the participants in our study. Despite the inability to qualify however, our findings revealed high unemployment rates among these cognitively able participants (i.e., IQ > 70), highlighting the need for tailored employment assistance regardless of cognitive abilities. Rather than focusing solely on those with profound disabilities, evidence-based supported employment models also may be critical for individuals across the spectrum of abilities.

### Factors Associated with Employment Status Among Autistic Youth

Executive functioning skills appear to play a crucial role in employment outcomes for autistic and non-autistic individuals during this transition period. The findings indicate that greater difficulties in executive functioning are associated with a lower likelihood of securing competitive employment. This finding aligns with prior research suggesting that deficits in working memory, inhibitory control, cognitive flexibility, planning, and self-awareness—core components of executive functioning—can create significant workplace challenges for autistic individuals (Demetriou et al., 2021; van den Bergh et al., 2014). Difficulties in these areas may contribute to problems with task initiation, organization, and adaptability, which can negatively impact job retention and performance (Bury et al., 2020; Woolard et al., 2021). Furthermore, poor executive functioning has been linked to lower levels of job satisfaction and increased workplace stress, potentially contributing to higher turnover rates (Bury et al., 2020).

In contrast to previous studies that identified verbal IQ and adaptive functioning as key predictors of employment (Hernández-Finch et al., 2023; Richardson & Norgate, 2015; Roux et al., 2013), our results did not find a significant relationship between these factors and employment status. Future research should further explore the mechanisms by which executive functioning influences workplace success and investigate potential interventions, such as cognitive training and executive function coaching, that could enhance employment outcomes for autistic individuals.

One limitation of this study is that the sample consisted of 99 participants all from California. This may limit the generalizability of our findings to the broader autistic population in other regions. Employment experiences and support services can vary widely based on geographic location, economic factors and policy differences; therefore, future research should aim to include more diverse and representative samples. We were unable to conduct subgroup analyses by educational status due to limited sample size; however, given that autistic individuals can remain in high school until age 21, future research should explore how educational stage impacts early employment outcomes.

In conclusion, our research provides insight into the multifaceted challenges autistic adults face in the workforce. High unemployment rates among autistic adults pose risks to their mental health, quality of life, and overall life satisfaction (Brouwers et al., 2023; Wehman et al., 2014, 2017). Financial instability resulting from unemployment limits their ability to achieve financial autonomy and live independently (Sosnowy et al., 2018). This challenge extends beyond the individual, affecting familial dynamics and increasing parental stress, as research indicates that at least 60% of autistic individuals continue to reside with their parents into adulthood (Levy & Perry, 2011). Additionally, unemployment among autistic adults has broader societal implications, contributing to economic dependency on social support systems (Hedley et al., 2016; Hendricks, 2010). Our findings highlight the importance of addressing executive functioning challenges, which emerged as a significant predictor of employment outcomes. Difficulties with cognitive flexibility, planning, and self-regulation may impact job acquisition and retention, underscoring the need for targeted interventions such as executive function coaching and workplace accommodations. Furthermore, our results emphasize the role of personal connections in job acquisition, which were found to be just as effective as supported employment services. This suggests that employment programs should actively involve families and social networks to expand job opportunities.

The findings from this study highlight the need for continued research on the effectiveness and long-term impact of services aimed at improving employment outcomes for autistic youth. While programs like WAI provide a valuable introduction to early employment, future research should examine how to expand and diversify these experiences to better support sustained, competitive, integrated, and meaningful employment. It is also essential to explore how educational and vocational systems can adapt their services to meet the evolving needs of autistic individuals during the transition to adulthood. To improve long-term outcomes, efforts should focus on developing and expanding supported employment services, promoting access to a wider range of job opportunities beyond entry-level positions, and integrating executive function support into vocational training. Additionally, fostering positive family involvement in job development may empower parents to better assist autistic job seekers (Carter et al., 2023; Schwartzman et al., 2023). Addressing these challenges is not only critical for the well-being and independence of autistic adults but also for fostering a more inclusive and supportive society.

## Supplementary Material

Supplementary Material

**Supplementary Information** The online version contains supplementary material available at https://doi.org/10.1007/s10803-025-07001-9.

## Figures and Tables

**Table 1:** Characterization of the sample

Characteristic	Non-autistic		Autistic		Independent t test			
	N	Mean (SD)	N	Mean (SD)	t (df)	p value	95% CI	Cohen’s d

Age	51	19.8 (1.5)	48	19.5 (1.5)	0.74 (97)	0.230	[− 0.374, 0.820]	0.16
FSIQ	51	109.6 (11.7)	48	102.0 (14.4)	2.92 (97)	0.002*	[2.460, 12.917]	0.59
VIQ	51	105.8 (13.2)	48	96.9 (15.7)	3.09 (97)	0.001*	[3.210, 14.726]	0.62
PIQ	51	111.1 (12.6)	48	107.0 (15.5)	1.46 (97)	0.074	[− 1.486, 9.760]	0.29
GEF	51	46.1 (10.0)	48	55.2 (10.9)	− 4.35 (97)	0.001*	[− 13.324, − 4.975]	− 0.88
GAC	48	111.5(11.7)	45	85.7 (16.0)	8.94 (91)	0.001*	[20.112, 31.596]	1.86
ADOS-2			48	8.1 (1.7)				

Ethnicity/race	*N*	%	*N*	%

White	24	47	22	46
Mixed	14	27	19	40
Asian	8	16	3	6
Latino/Hispanic	4	8	2	4
African American	1	2	2	4

	*N*	%	*N*	%	Df	*X* ^*2*^	*p* value	Cramer’s *V*

*Education level*								
Enrolled in high school	**3**	**6**	**14**	**29**	**2**	**15.29**	**0.001** *	**0.39**
Completed high school	**6**	**12**	**12**	**25**				
Post-secondary	42	82	22	46				
*Employment status* ^a^								
Employed	44	86	32	67	1	5.33	0.021*	0.23
Unemployed	7	14	16	23				
*Strict employment status* ^b^								
Employed	40	78	24	50	1	8.75	0.003*	0.30
Unemployed	11	22	24	50				

*FSIQ* Full-Scale Intelligence Quotient, *VIQ* Verbal Intelligence Quotient, *PIQ* Performance Intelligence Quotient, *GEF* Global Executive Functioning, *GAC* Global Adaptive Composite

a Employment status: paid and unpaid work experiences were counted as employed

b Strict employment status: paid and unpaid work experiences were not counted as employed

* Significant at *p* <.05 level

**Table 2:** Characterization of work experiences

Characteristics	Work experience 1				Work experience 2				Work experience 3				Work experience 4			
	Non-autistic (*n* = 42)		Autistic (*n* = 31)		Non-autistic (*n* = 12)		Autistic (*n* = 9)		Non-autistic (*n* = 3)		Autistic (*n* = 2)		Non-autistic (*n* = 1)		Autistic (*n* = 0)	
	*n*	*%*	*n*	*%*	*n*	*%*	*n*	*%*	*n*	*%*	*n*	*%*	*n*	*%*	*n*	*%*

*Job setting*																
Fully inclusive	42	100	28	90	12	100	9	100	3	100	2	100	1	100	–	–
Supported employment group	–	–	–	–	–	–	–	–	–	–	–	–	–	–	–	–
Work activity programs	–	–	3	10	–	–	–	–	–	–	–	–	–	–	–	–
Sheltered workshop	–	–	–	–	–	–	–	–	–	–	–	–	–	–	–	–
*Employment support*																
Independent work	42	100	24	77	12	100	9	100	3	100	2	100	1	100	–	–
Group employment classes	–	–	2	7	–	–	–	–	–	–	–	–	–	–	–	–
Job coach	–	–	4	13	–	–	–	–	–	–	–	–	–	–	–	–
Other (supervisor)	–	–	1	3	–	–	–	–	–	–	–	–	–	–	–	–
*Job duration*																
1 month or less	1	3	4	13	–	–	–	–	–	–	–	–	–	–	–	–
2–3 months	6	16	10	33	1	8	3	42	–	–	–	–	–	–	–	–
4–5 months	4	11	4	13	1	8	1	17	–	–	1	50	–	–	–	–
6–7 months	6	16	3	10	3	25	3	42	–	–	–	–	–	–	–	–
8–9 months	1	3	–	–	2	17	–	–	1	33	–	–	–	–	–	–
10–11 months	1	3	–	–	–	–	–	–	–	–	–	–	–	–	–	–
1 year	5	14	2	7	–	–	–	–	1	33	–	–	–	–	–	–
More than 1 year	13	35	7	23	4	33	–	–	1	33	1	50	–	–	–	–
Ongoing	–	–	–	–	1	8	–	–	–	–	–	–	1	100	–	–
Missing Information	5	–	1	–	–	–	2	–	–	–	–	–	–	–	–	–
*Weekly work hours*																
Less than 10 h	8	19	10	32	2	17	4	44	–	–	1	50	–	–	–	–
10–19 h (semi part-time position)	14	33	11	36	1	8	1	11	1	33	–	–	–	–	–	–
20–39 h (part–time position)	13	31	9	29	4	33	2	22	–	–	1	50	–	–	–	–
40 h or more (full–time position)	7	17	1	3	5	42	2	22	2	67	–	–	1	100	–	–
*Job sector– entry–level jobs*																
Building and ground cleaning and maintenance	–	–	1	3	1	8	2	22	–	–	–	–	–	–	–	–
Food preparation and serving	9	21	4	13	3	25	1	11	1	33	–	–	–	–	–	–
Office and administrative support	3	7	3	10	–	–	–	–	–	–	–	–	–	–	–	–
Production	–	–	1	3	–	–	–	–	–	–	–	–	–	–	–	–
Sales/retail*	6	14	16	52	2	17	2	22	–	–	1	50	–	–	–	–
Transportation and material moving	2	5	2	7	–	–	–	–	1	33	–	–	–	–	–	–
*Job sector—career-track jobs*																
Architecture and engineering	–	–	–	–	2	17	–	–	–	–	–	–	1	100	–	–
Arts, design, entertainment, sports, and media	5	12	1	3	–	–	–	–	–	–	–	–	–	–	–	–
Community and social services	3	7	–	–	–	–	–	–	–	–	–	–	–	–	–	–
Computer and mathematical	1	2	–	–	1	8	–	–	1	33	–	–	–	–	–	–
Educational instruction and library	6	14	3	10	–	–	4	44	–	–	1	50	–	–	–	–
Healthcare support	1	2	–	–	1	8	–	–	–	–	–	–	–	–	–	–
Life, physical, and social sciences	5	12	–	–	1	8	–	–	–	–	–	–	–	–	–	–
Management	1	2	–	–	–	–	–	–	–	–	–	–	–	–	–	–
Safety & inspection officer	–	–	–	–	1	8	–	–	–	–			–	–		
*Job acquisition method*																
Competitive process	40	95	15	48	11	92	6	67	3	100	2	100	1	100	–	–
Personal connections	2	5	3	10	–	–	–	–	–	–	–	–	–	–	–	–
Supported employment program services	–	–	5	16	–	–	1	11	–	–	–	–	–	–	–	–
Other	–	–	8^a^	26	l^b^	8	2^a^	22	–	–	–	–	–	–	–	–

a WorkAbility I Program facilitated these positions

b Started with an internship

* Most autistic participants were working in retail jobs, specifically stocking shelves, cleaning, or bagging groceries at supermarkets and grocery stores

**Table 3:** Logistic regression predicting employment status for first job experience (unpaid and paid work experiences)

Variables	Estimate	SE	OR	Wald X^2^	*p* value	CI	
						Lower bound	Upper bound

M_0_ (intercept)	1.17	0.24	3.23	23.06	0.001	0.69	1.65
M_1_ (intercept)	− 4.32	5.77	0.01	0.56	0.454	− 15.62	6.98
Dx (autistic)	− 0.92	0.76	0.40	1.47	0.225	− 2.41	0.57
FSIQ	− 0.41	0.42	0.67	0.96	0.327	− 1.23	0.41
VIQ	0.21	0.24	1.23	0.73	0.392	− 0.27	0.69
PIQ	0.22	0.22	1.24	0.94	0.334	− 0.22	0.65
GAC	0.03	0.02	1.03	2.26	0.133	− 0.01	0.08
GEF	0.03	0.03	1.04	0.72	0.396	− 0.03	0.09
M_2_ (intercept)	− 7.66	7.58	4.73	1.02	0.312	− 22.51	7.20
Dx (autistic)	− 0.86	0.78	0.42	1.22	0.269	− 2.39	0.67
FSIQ	− 0.53	0.43	0.59	1.46	0.227	− 1.38	0.33
VIQ	0.28	0.26	1.32	1.21	0.272	− 0.22	0.78
PIQ	0.28	0.23	1.32	1.43	0.232	− 0.18	0.73
GAC	0.03	0.02	1.07	1.17	0.279	− 0.02	0.07
GEF	0.03	0.03	1.03	0.88	0.349	− 0.03	0.09
Ed. Level	0.79	0.60	2.19	1.74	0.187	− 0.38	1.95
Age	0.09	0.183	1.10	0.26	0.611	− 0.27	0.45

Employment status level “Employed” was coded as class 1

*SE* standard error, *OR* odds ratio, *CI* confidence interval, *FSIQ* Full-Scale Intellectual Quotient, *VIQ* Verbal Intellectual Quotient, *PIQ* Performance Intellectual Quotient, *GAC* General Adaptive Composite, *GEF* Global Executive Functioning

**Table 4:** Logistic regression predicting employment status for first job experiences (without unpaid work experiences)

Variables	Estimate	SE	OR	Wald X^2^	*p* value	CI	
						Lower bound	Upper bound

M_0_ (intercept)	0.55	0.22	1.74	6.55	0.010	0.13	0.97
M_1_ (intercept)	− 7.91	5.38	3.65	2.17	0.141	− 18.45	2.62
Dx (autistic)	− 1.14	0.69	0.32	2.74	0.098	− 2.49	0.21
FSIQ	− 0.39	0.38	0.68	1.04	0.308	− 1.14	0.36
VIQ	0.20	0.22	1.22	0.77	0.380	− 0.24	0.64
PIQ	0.20	0.21	1.23	0.98	0.323	− 0.20	0.61
GAC	0.05	0.02	1.05	5.99	0.014*	0.01	0.10
GEF	0.06	0.03	1.07	5.00	0.025	0.01	0.12
M_2_ (intercept)	− 17.77	7.79	1.91	5.21	0.022	− 33.03	− 2.51
Dx (autistic)	− 1.09	0.75	0.34	2.11	0.146	− 2.55	0.38
FSIQ	− 0.75	0.43	0.47	3.09	0.079	− 1.59	0.09
VIQ	0.41	0.25	1.51	2.71	0.100	− 0.08	0.90
PIQ	0.40	0.23	1.49	2.97	0.085	− 0.05	0.85
GAC	0.04	0.02	1.04	3.05	0.081	− 0.01	0.09
GEF	0.07	0.03	1.08	6.19	0.013*	0.02	0.13
Ed. level	1.72	0.60	5.58	8.28	0.004*	0.55	2.89
Age	0.25	0.18	1.28	1.88	0.170	− 0.11	0.61

Employment status level “Employed” was coded as class 1

*SE* standard error, *OR* odds ratio, *CI* confidence interval, *FSIQ* Full-Scale Intellectual Quotient, *VIQ* Verbal Intellectual Quotient, *PIQ* Performance Intellectual Quotient, *GAC* General Adaptive Composite, *GEF* Global Executive Functioning

* Significant at *p* <.05 level

## Footnotes

1 The term youth typically refer to individuals between the ages of 15 and 24 years, the time when individuals are transitioning from the higher levels of education to employment (Bersaglio, Enns, & Kepe, 2015), although some extend this period to age 35.

2 WorkAbility I: A California Transition Program - Services & Resources (CA Dept of Education).
