# Supplementary material for: Early Employment Outcomes in Autistic and Non-autistic Youth: Challenges and Opportunities
Source: J Autism Dev Disord. Author manuscript; Available in PMC 2025 Dec 14. (PMC12702462; doi:10.1007/s10803-025-07001-9)
Supplement: Supplementary Material [file NIHMS2122833-supplement-Supplementary_Material.docx]

**Supplementary Information**

**Title:** Early Employment Outcomes in Autistic and Non-Autistic Youth: Challenges and Opportunities

**Journal:** Journal of Autism and Developmental Disorders

**Authors:** Jo A. Yon-Hernández^1^, Catherine Gonzales^1^, Surina Bothra^1^, Kali Kecskemeti^1^, Ana-Maria Iosif^2^, Yukari Takarae^1^, Steve Ruder^3^, Susan R. McGurk^4^, Kim T. Mueser^4^, & Marjorie Solomon^1^

**Corresponding author:** Jo A. Yon-Hernández, PhD**.** Email: [jyonh@health.ucdavis.edu.](mailto:jyonh@health.ucdavis.edu.) Address: 2825 50^th^ Street, Sacramento, CA 95817

**Measures**

*Global Functioning: Role Scale (GF: Role) (Carrión et al., 2018; Cornblatt et al., 2015)*

As part of the adaptation of the GF:Role scale for this study, we refined prompts and anchors to better capture autism-specific role and social challenges and ensure age-appropriateness. Expert consensus informed these adaptations to enhance the scale’s relevance and utility for the study population. Specific questions to guide the rating of the GF:Role scale are provided below. Ratings should consider changes in role functioning over the previous year (capturing both highest and lowest levels of functioning) as well as current functioning within the past month. Below, additional prompts included in this adaptation are highlighted in **bold**.

**GF:Role**

1. How do you spend your time during the day? **Query about paid work, volunteer jobs, internships, work group, sheltered workshop participation.**

2. If currently working **in paid or unpaid work**:

1. Where do you work? What are your job responsibilities?
2. How many hours a week do you work?
3. How long have you been in your current job?
4. Have you had any recent changes in your job status (eg, lost job, stopped working, changed position, or workload)?
5. Do you usually need assistance or regular supervision? Explain how often you need help and if there are tasks you cannot do without help. Do you ever have trouble keeping up? Are you able to catch up if you fall behind?
6. Have you received any comments (positive or negative) or formal reviews regarding your performance? Have others pointed out things that you have done well or poorly?

3. If currently attending school:

1. What type of school do you attend? (**General education high school, nonpublic school, residential/hospital school, community college, four-year college**)
2. How long have you been at this school? Have you had any recent changes in your school placement?
3. Have you ever been in special education classes or other non-general education classes either now or previously in high school or college?
4. Do you receive any extra help or accommodations in your classes (e.g. tutoring or extra help in school or after school; extra time to take tests or are you able to leave the classroom to take tests in a quiet place)?
5. Do you have trouble keeping up with your coursework? Are you able to catch up if you fall behind?
6. How are your grades? Are you failing any classes?
7. **Is it likely you will be able to graduate from your current school?**
8. **Do you intend to get more advanced training after college graduation?**

4. If a homemaker:

1. What are your responsibilities around the house related to housekeeping and caring for the family?
2. How long have you been in charge of the home?
3. How many hours per week do you spend working on household tasks?
4. Are you able to keep up with the demands of your household? Do you ever fall behind? If so, are you able to catch up or do you need others’ help?
5. **Do you spend anytime volunteering in the community? If so, where do you volunteer? What do you do? How much time does this take? Do you hold a leadership role where you volunteer?**

Additionally, to the prompt additions, we also proposed the following adaptations to the original GF:Role rating scale (Cornblatt et al., 2007).

| GF: Role Scale | |
| --- | --- |
| **Score** | **Superior role functioning** |
| **10** | **Original:** Independently maintains superior functioning in demanding roles. Obtains only superior performance evaluations at competitive work placement. Obtains all A’s in mainstream school. Generates, organizes, and completes all homemaking tasks with ease.  **Proposed:** The individual may attend mainstream high school or college with no support. They get As in all classes. If they have completed school, the individual holds a full-time job at a competitive wage and has been in this role or field for several years. They perform at a superior level in a highly demanding leadership role. Alternatively, the individual may be a full-time homemaker who volunteers in the community, where they hold leadership roles. The individual has demonstrated the ability to live independently in the community. They may also be a spouse and parent and are able to perform these roles very well. |
|  | **Above average role functioning** |
| **9** | **Original:** Independently maintains very good functioning in demanding roles. Rarely absent or unable to perform. Obtains good to superior performance evaluations at competitive work placement. Obtains grades in A and B range in all courses in mainstream school. Generates, organizes, and completes all homemaking tasks.  **Proposed:** The individual may attend high school or community college successfully, with no support. They get grades in the A and B range in mainstream school. If they have graduated, the individual holds a full-time job at a competitive wage and has been in this role consistently for a period of at least a year. Alternatively, the individual may be a full-time homemaker who volunteers in the community. They perform at a high level in a demanding role. The individual has demonstrated the ability to live independently in the community. They may also be a spouse and parent and are able to perform these roles competently. |
|  | **Good role functioning** |
| **8** | **Original:** Independently maintains good role functioning in demanding roles. Occasionally falls behind on tasks but always catches up; obtains satisfactory performance evaluations at competitive work placement; obtains grades of C and above in mainstream school; occasional difficulty generating or organizing homemaking tasks; or maintains above average performance with minimal support (eg, tutoring, reduced academic course load at 4-year university, attends community college, may receive additional guidance at work less than 1–2 times a week). Receives As and Bs, good work/school evaluations, and completes all tasks with this level of support.  **Proposed:** The individual may attend high school or community college successfully, with no support. They get grades in the A and B range in mainstream school. If they are a student, they may hold a paid part-time job. If they have graduated, they may hold a full-time job at a competitive wage without more than normal levels of support from co-workers and supervisors. Alternatively, the individual may be a full-time homemaker. Occasionally they may fall behind at work or at homemaking or make a mistake, but they generally can self-correct and catch up. The individual has demonstrated the ability to live independently, relying only on natural supports (parents, peers, neighbors, etc.) to sustain a residence in the community, and now can afford to do so. They are able to drive or use public transportation on their own. |
|  | **Mild impairment in role functioning** |
| **7** | **Original:** Mildly impaired functioning in demanding roles independently. Frequently behind on tasks or unable to perform; frequently obtains poor performance evaluations at competitive work placement or grades of Ds or better in mainstream school; frequent difficulty generating or organizing homemaking tasks; or maintains good performance with minimal support (e.g., minimal accommodations in general education classroom, receives additional guidance/support at work 1–2 times a week). Receives Cs or higher, satisfactory work/school evaluations, and completes most homemaking tasks with this level of support.  **Proposed:** The individual can attend high school or community college successfully, with little support. They obtain grades of C or higher. If the individual is older, they may have completed college, but currently may not be employed because they have been unable to get a job or to be accepted at a graduate school program. Alternatively, they may have had a job at a competitive salary but have been laid off. The individual has demonstrated the ability to live independently, relying only on natural supports (parents, peers, neighbors, etc.) to sustain a residence in the community, although they may not currently be able to afford to do so. They are able to drive or use public transportation on their own. |
|  | **Moderate impairment in role functioning** |
| **6** | **Original:** Moderate impairment independently. May receive occasional F in mainstream courses, persistently poor performance evaluations at competitive work placement; may change jobs because of poor performance, persistent difficulty generating, or organizing homemaking tasks; or requires partial support (some resource or special education courses, receives guidance/support at work 2+ times per week). May require less demanding or part-time jobs and/or some supervision in home environment but functions well or adequately given these supports (may fall behind but eventually completes assigned tasks, obtains satisfactory evaluations at work or passing grades in school).  **Proposed:** The individual can attend high school or community college successfully, with little support. Occasionally, they get poor grades due to deficits in organizational skills, but they are expected to graduate. If they already have graduated from high school, they may have a minimally demanding part-time job or may be a homemaker. This individual is not yet able to live independently without support, but they participate meaningfully in housework in the place where they live. This includes identifying new chores that need to be completed. They also are able to drive or use public transportation on their own. |
|  | **Serious impairment in role functioning** |
| **5** | **Original:** Serious impairment independently. Failing multiple courses in mainstream school, may lose job, or unable to complete most homemaking tasks independently; or in entirely special education classes, requires less demanding job/daily support or guidance, may require vocational rehabilitation, and/or some supervision in home environment but maintains ‘‘above average’’ performance—receives As and Bs, good evaluations at work/school, completes all tasks.  **Proposed:** Individual may attend a regular high school or community college and take a minimally reduced course load and/or receive accommodations from the student disability center. Individual participates in a vocational program such as an enclave, work group or sheltered workshop and has some income. They need little supervision in the program and are able to use public transportation or drive. Alternatively, with the help of the student disability center or other special education service, they may attend 2 or 4-year community college with the goal of obtaining a regular undergraduate degree. They need considerable support to stay organized and receive poor grades at times. They are not yet able to live independently but participate in a meaningful way in housework in the place where they live. |
|  | **Major impairment in role functioning** |
| **4** | **Original:** Very serious impairment independently. All Fs in mainstream school or failing out of school; cannot obtain or hold independent job or unable to complete virtually any homemaking tasks independently; or adequate to good functioning with major support. Requires assisted work environment, entirely special education classes, nonpublic or psychiatric school, home schooling for the purpose of a supportive school environment, and/or supported home environment but functions adequately given these supports (may fall behind but completes assigned tasks, obtains satisfactory performance evaluations at work or passing grades).  **Proposed:** Individual may attend a regular high school or community college and take a reduced course load and/or receive accommodations from the student disability center. They also may participate in a program that promotes vocational skills such as an enclave, work group, or a sheltered workshop. They require some supervision in this job. They may have unpaid internships or volunteer jobs in the community for < = 5 hrs/week. They do not yet live independently but may use public transportation on their own. They also participate in a meaningful way in housework in the place where they live. |
|  | **Marginal ability to function** |
| **3** | **Original:** Impaired functioning with major support. Requires supported work environment, entirely special education classes, nonpublic or psychiatric school, home schooling for the purpose of a supportive school environment, and/or supported home environment but functions poorly despite these supports (persistently behind on tasks, frequently unable to perform, obtains poor performance evaluations at work or fails courses at school).  **Proposed:** Individual does not work for pay, attend regular (full load or graded) high school or college classes or live independently. However, he or she attends a program that promotes vocational skills such as an enclave, work group, or a sheltered workshop. They are able to help with simple household chores. They may have an unpaid internship or volunteer job in the community for < = 2 hrs/week. |
|  | **Inability to function** |
| **2** | **Original:** Disabled but participates in structured activities. On disability or equivalent non-independent status. Not working for pay, attending classes for grades, or living independently. Spends 5 or more hours a week in structured role-related activities (eg, residential treatment, volunteering, tutoring, sheltered work programs).  **Proposed:** Individual does not work for pay, attend regular (full load or graded) high school or college classes or live independently. They spend < = 2 hours/week in a structured role-related activity in the community, such as a volunteer job or internship. They also may attend a day program but earn no money. They may be able to help with simple household chores. |
|  | **Extreme role dysfunction** |
| **1** | **Original:** Severely disabled. On disability or equivalent non independent status. Not working for pay, attending classes for grades, or living independently. Spends fewer than 5 hours a week in structured role-related activities.  **Proposed:** Individual does not work for pay, attend regular (full load or graded) high school or college classes or live independently. They spend no time in a structured role-related activity in the community. |

*Coding of Open-Ended Responses*

Open-ended questionnaire responses were systematically coded using a predefined classification scheme developed to capture key characteristics of participants’ employment experiences. The coding process included the following categories:

1. *Type of Job Setting.* Participants' descriptions of their job environments were classified into one of the following categories: (a) Fully Inclusive Setting: Individuals worked in typical community settings with regular interaction alongside coworkers without disabilities. (b) Supported Employment Group (Crews): Individuals worked in small groups of people with disabilities, typically receiving ongoing job support. (c) Work Activity Programs (Sheltered Workshops): Individuals worked in segregated environments without integration with typically developing peers and were often paid below minimum wage. (d) Other: Any job setting that did not fall under the above categories.
2. *Type of Employment Support.* Participants were asked to describe the nature of any employment support they received. Responses were coded into: (a) Independent Work: The individual worked without formal support. (b) Group Employment Classes: Participation in structured, group-based classes focused on developing employment-related skills. (c) Job Coach: The presence of an individual providing support with day-to-day job responsibilities and challenges. (d) Other – Any form of support not covered by the above classifications.
3. *Job Acquisition Method*. Participants’ methods of securing employment were categorized as: (a) Competitive Process: Job obtained through a typical application and interview process based on merit and qualifications. (b) Personal Connections: Employment obtained through family, friends, or other informal networks. (c) Supported Employment Program or Agency: Jobs arranged through state/federal programs or agencies offering job development and coaching. (d) Other: Methods of job acquisition that did not align with the categories above.
4. *Job Sector.* Participants described the nature of their jobs, which were classified into one of two broad categories—***Entry-Level Jobs*** or ***Career-Track Jobs***—based on typical educational requirements, training, and potential for career advancement. Classification was informed by the U.S. Bureau of Labor Statistics’ Standard Occupational Classification (SOC) system and occupational outlook criteria (U.S. Bureau of Labor Statistics, 2024).
5. *Job Duration.* Participants reported how long they remained in each job. Responses were categorized into the following durations: (a) 1 month or less; (b) 2–3 months; (c) 4–5 months; (d) 6–7 months; (e) 8–9 months; 10–11 months; (g) 1 year; (h) More than 1 year; and (i) Ongoing at the time of data collection.
6. *Weekly Work Hours*. Participants indicated how many hours per week they typically worked in each job. Work hours were grouped into the following categories: (a) Less than 10 hours; (b) 10–19 hours (Semi Part-Time Position); (c) 20–39 hours (Part-Time Position); (d) 40 hours or more (Full-Time Position).

References

Cornblatt, B. A., Auther, A. M., Niendam, T., Smith, C. W., Zinberg, J., Bearden, C. E., & Cannon, T. D. (2007). Preliminary findings for two new measures of social and role functioning in the prodromal phase of schizophrenia. *Schizophrenia bulletin*, *33*(3), 688–702. <https://doi.org/10.1093/schbul/sbm029>

U.S. Bureau of Labor Statistics. (2024). Occupational Outlook Handbook. https://www.bls.gov/ooh/
